# Cognitive estimation: Performance of patients with focal frontal and posterior lesions

**DOI:** 10.1016/j.neuropsychologia.2017.08.017

**Published:** 2018-07-01

**Authors:** Lisa Cipolotti, Sarah E. MacPherson, Sara Gharooni, Natasja van-Harskamp, Tim Shallice, Edgar Chan, Parashkev Nachev

**Affiliations:** a Department of Neuropsychology, National Hospital for Neurology and Neurosurgery, London, UK; b Dipartimento di Scienze Psicologiche, Pedagogiche e della Formazione, Università degli Studi di Palermo, Palermo, Italy; c Centre for Cognitive Ageing and Cognitive Epidemiology, University of Edinburgh, Edinburgh, UK; d Department of Psychology, University of Edinburgh, Edinburgh, UK; e Institute of Cognitive Neuroscience, University College London, UK; f International School for Advanced Studies (SISSA-ISAS), Trieste, Italy; g Institute of Neurology, UCL, UK & National Hospital for Neurology and Neurosurgery, London, UK

**Keywords:** CVA, cerebrovascular accident, GNT, Graded Naming Test, HC, healthy comparisons, IQ, Intelligence Quotient, LF, left frontal, NART, National Adult Reading Test, No, Number, PFC, prefrontal cortex, RAPM, Raven's Advanced Progressive Matrices, Cognitive estimation test, Prefrontal cortex, Executive functions, Fluid intelligence

## Abstract

The Cognitive Estimation Test (CET) is a widely used test to investigate estimation abilities requiring complex processes such as reasoning, the development and application of appropriate strategies, response plausibility checking as well as general knowledge and numeracy (e.g., Shallice and Evans, 1978; MacPherson et al., 2014). Thus far, it remains unknown whether the CET is both sensitive and specific to frontal lobe dysfunction. Neuroimaging techniques may not represent a useful methodology for answering this question since the complex processes involved are likely to be associated with a large network of brain regions, some of which are not functionally necessary to successfully carry out the CET. Instead, neuropsychological studies may represent a more promising investigation tool for identifying the brain areas necessary for CET performance. We recently developed two new versions of the CET (CET-A and CET-B; MacPherson et al., 2014). We investigated the overall performance and conducted an error analysis on CET-A in patients with focal, unilateral, frontal (n = 38) or posterior (n = 22) lesions and healthy controls (n = 39). We found that frontal patients’ performance was impaired compared to healthy controls on CET. We also found that frontal patients generated significantly poorer estimates than posterior patients on CET-A. This could not be explained by impairments in fluid intelligence. The error analyses suggested that for CET-A, *extreme* and *very extreme* responses are impaired following frontal lobe damage. However, only *very extreme* responses are significantly more impaired following frontal lobe than posterior damage and so represent a measure restricted to frontal “executive” impairment, in addition to overall CET performance.

## Highlights

• The prefrontal cortex (PFC) is critical for performance on the CET.
• CET-A overall scores and *very extreme* responses are affected and restricted to PFC damage.
• CET-A is an ‘executive’ task for which impairment cannot be accounted by fluid intelligence.

## 1 Introduction

Cognitive estimation tasks require the ability to generate responses to questions for which exact answers are not readily available. These estimation tasks assess an important form of problem-solving which is often required in everyday activities (e.g., estimating your next shopping bill or the size of an item of clothing you should buy as a gift). Estimation relies on complex processes such as reasoning, the development and application of appropriate strategies, response plausibility checking as well as general knowledge and numeracy (e.g., Shallice and Evans, 1978; MacPherson et al., 2014). Patients, who experience brain damage, often involving the frontal lobes, are reported to have impaired judgement and problem-solving abilities and generate estimates that are considered to be bizarre. For example, Shallice and Evans (1978) described a patient who, following a large right frontal lesion caused by an explosion, showed a severe impairment in producing adequate cognitive estimates. When he was asked, ‘*What is the height of the highest building in London?*’, he replied “18,000 to 20,000 feet” (approximately 5500-6000 m). Strikingly, the patient did not appear to realize that his answers were bizarre and instead continued to justify them, even when pressed about the appropriateness of the responses.

Shallice and Evans (1978) developed the Cognitive Estimation test (CET) to formally investigate estimation abilities in frontal patients. The original CET comprised of 15 questions and has long since been used to assess estimation abilities in both clinical and research settings. Several different versions of the CET have been developed (e.g., Brand et al., 2003; Bullard et al., 2004) and studies have reported normative data for these different CET versions (e.g., Axelrod and Millis, 1994; Della Sala et al., 2003; Scarpina et al., 2015, for a review of the different CET versions see Wagner et al., 2011). We have recently developed two new 9-item parallel versions of the CET (i.e., CET-A and CET-B) with the aim of providing more up-to-date items that can be administered in different countries, on more than one occasion (MacPherson et al., 2014).

The CET is widely considered to be a test of executive function and has been included as such in several handbooks of neuropsychology (e.g., Denes and Pizzamiglio, 1999; Strauss et al., 2006; Gurd et al., 2010). Executive functions refer to a variety of general purpose control mechanisms thought to modulate and organize more basic cognitive sub-processes to achieve goal-oriented behaviour (e.g., Stuss and Levine, 2002). In order to provide appropriate estimates, individuals need to identify and select the appropriate way of thinking or interpreting information, retrieve and manipulate particular details or estimates, monitor how appropriate their response is and finally repeat this procedure if a better estimate is required. However, there are also studies that do not support the notion that the CET assesses executive abilities, as performance on the CET and other executive measures such as verbal fluency, the Trail Making Test, the Wisconsin Card Sorting Test or the Frontal Assessment Battery do not significantly correlate (e.g., Spreen and Strauss, 1998; Appollonio et al., 2005; Spencer and Johnson-Greene, 2009; Barabassy et al., 2010; D’Aniello et al., 2015). Recently D’Aniello and colleagues (2015) suggested that the CET, “… may be considered a useful instrument for the assessment of crystallized intelligence and of cognitive reserve…” but it is not a “…specific measure of executive functions.” (p. 3).

Executive functions are thought to be mediated primarily by the frontal lobes (e.g., Stuss and Levine, 2002). However, the precise nature of the frontal lobes’ contribution to executive abilities remains poorly understood (e.g., Hornberger and Bertoux, 2015). Several theories suggest that component processes of executive functions rely on specific subregions within the prefrontal cortex (e.g., Stuss and Alexander, 2007; Shallice et al., 2008; Petrides, 2005). In contrast, some other theories suggest that a large fronto-parietal network, named the *multiple-demand network*, carries out general control processes that match the requirements of the task being undertaken, independently of the type of information being processed (e.g., Duncan, 2001; Miller and Cohen, 2001). This putative network has been proposed to be the seat of general fluid intelligence or Spearman's *g* (e.g., Spearman, 1904, Spearman, 1927; Woolgar et al., 2010), which is known to positively correlate with performance on tests of executive function and is impaired following frontal lesions (Duncan et al., 1995).

These different theories have important implications for understanding frontal patients’ impairments on executive tests, such as the CET. In an influential paper, Roca et al. (2010) argued that fluid intelligence is a substantial contributor to frontal-executive deficits. The authors reported that impairments in fluid intelligence can explain executive impairments on several well-known ‘executive tests’ such as the Wisconsin Card Sorting Task or letter fluency. In frontal patients, after partialling out the contribution of fluid intelligence, impairments remained only for a small number of ‘frontal’ tasks. This finding has raised questions regarding the diagnostic significance of executive tests. However, very few studies have investigated whether executive impairments in frontal patients can be explained by a loss in fluid intelligence. Recently, Cipolotti et al. (2016) have reported that impairments on the Hayling and Stroop tests in frontal patients cannot be fully explained by fluid intelligence. Therefore, it remains important to establish the extent to which a loss of fluid intelligence can account for CET impairments in frontal patients.

It also remains important to establish whether the CET is a test sensitive and specific to frontal lobe damage. It has been reported that CET performance is impaired in a variety of neurological conditions such as stroke (Shoqeirat et al., 1990), Alzheimer's disease (Della Sala et al., 2004), frontotemporal dementia and corticobasal syndrome (Bisbing et al., 2015), Korsakoff's syndrome (Brand et al., 2003), Huntington's disease (Brandt et al., 1988) and traumatic brain injury (Schretlen, 1992) and psychiatric conditions such as schizophrenia (e.g., Roth et al., 2012; Gansler et al., 2014). However, these studies do not allow us to determine whether the CET is a test specific to frontal lobe damage.

Surprisingly, only a handful of focal lesion studies have specifically investigated the frontal specialization of the CET. The evidence reported so far is inconsistent or sparse. Shallice and Evans (1978) first reported that patients with unilateral left or right anterior lesions produced significantly more bizarre answers than patients with posterior lesions on the CET. Similarly, Smith and Milner (1984) found that right unilateral frontal lobectomy patients (n = 12) made significantly more errors than healthy controls (HC), and left and right temporal lobectomy patients on a price estimation task. However, no CET impairments were detected in the small left frontal group (n = 7) who had smaller lesions than the right frontal group. In contrast, Taylor and O'Carroll (1995) did not find a significant difference between anterior and posterior patients performing the CET. Stanhope et al. (1998) reported a significant difference between the performance of frontal patients (n = 9) with stereotactic subcaudate tractotomy for treatment of intractable affective disorders and HCs. However, they found no significant difference between frontal, diencephalic and temporal lesion patients.

Notably, from these studies it remains unclear whether the reported lesions were indeed confined to the frontal or the posterior lobes. For example, approximately 50% of the anterior patients reported by Shallice and Evans (1978) had large tumours extending beyond the frontal lobes (i.e., fronto-temporal or fronto-parietal lesions). Almost half of Taylor and O'Carroll (1995)’s anterior patients (7 out 15) suffered a head injury whilst the posterior group included patients with head injury and focal cortical atrophy. The diencephalic and temporal patients reported by Stanhope et al. (1998) had alcoholic Korsakoff syndrome or herpes encephalitis or anoxia. More recently, MacPherson and colleagues (2014) reported that a group of patients with focal lesions confined to the frontal lobes based on clinical CT or MRI scans, performed more poorly than HCs on both versions of the CET. However, no data from patients with posterior lesions were included. Hence, it remains unknown if the two new CET versions are specific to frontal lobe lesions.

Despite rapid advancements in neuroimaging methodologies such as PET, fMRI, EEG and MEG for identifying the brain regions associated with specific cognitive processes, to our knowledge, no neuroimaging study has examined the neural correlates of the CET. This is perhaps not surprising. Complex processes such as those involved in cognitive estimation are likely to be associated with the activation of large brain networks. Critically, the activation of brain areas in a functional imaging study does not necessitate that these areas are functionally necessary to successfully carry out the task (e.g., Gilaie-Dotan et al., 2015). Hence, neuroimaging techniques may not represent a useful methodology for the investigation of cognitive estimation. Instead, neuropsychological studies may represent a more promising investigation tool for identifying the brain areas that are necessary for CET performance.

The aim of the current study was to investigate these theoretical and anatomical issues in relation to one of our two recently developed versions of the CET (CET A) in patients with focal, unilateral, frontal or posterior lesions and HCs. We grouped together focal non-traumatic frontal lesions due to tumour or stroke, as our previous work has shown that the grouping of patients with frontal lesions due to low- and high-grade glioma, meningioma or stroke (n = 100) is a pragmatic and justified methodological approach when examining frontal-executive functions (Cipolotti et al., 2015). We previously compared 100 frontal patients with four different types of aetiology- vascular, high-grade gliomas, low-grade gliomas and meningiomas- on four frontal executive tasks (Advanced Progressive Matrices, Stroop Colour-Word Test, Letter Fluency-S; Trail Making Test Part B; Cipolotti et al., 2015). We found strong behavioral effects of age and premorbid cognitive abilities on performance of the frontal tests. However, on only one test – Trail-Making Part B - was a significant difference between aetiologies obtained when age was partialled out in an ANCOVA. Critically, the significance did not survive Bonferroni correction, as there was no reason to consider Trail-Making, which later research shows not to be specific for frontal lesions (Chan et al., 2014), to be more susceptible to differences in aetiology than the other three tests. We therefore conclude that it is acceptable practice to mix aetiologies to overcome the great variability in the population under study. In our sample of frontal and non-frontal patients, we investigated whether: 1. CET A is affected by frontal lobe damage (i.e., compared to HCs), and 2. How restricted CET A is to frontal lobe damage (i.e., compared to posterior patients). In addition, we analysed the nature of the errors made by our patients. This is a source of evidence that it is generally not available from neuroimaging methods. We also investigated whether cognitive estimation is supported by a common, general process such as fluid intelligence, based on multiple demand regions (e.g., Roca et al., 2010).

## 2 Methods and materials

### 2.1 Participants

Forty-four patients with focal non-traumatic frontal lesions and 43 patients with focal non-traumatic posterior lesions were recruited from the Neuropsychology Department of the National Hospital for Neurology and Neurosurgery, Queen Square, London. Inclusion criteria for the study were (a) the presence of a lesion due to stroke or tumour based on a clinical CT or MRI scan, (b) >70% of the total lesion in the frontal lobes or in the posterior lobes, (c) ability to consent and complete over 75% of neuropsychological and experimental tasks, including CET A, (d) aged between 18 and 80 years, (e) no gross language disturbances, i.e. >5th %ile cut-off on the Graded Naming Test (McKenna and Warrington, 1983), and (f) absence of psychiatric disorders, history of alcohol or substance abuse or previous neurological disorders.

Application of the inclusion criteria resulted in a final sample of 34 frontal patients (left-sided lesion n = 15, right-sided lesion n = 19) and 19 posterior patients (left-sided lesion n = 8, right-sided lesion n = 11). The aetiologies of the lesions were either stroke (n = 17; 9 frontal, 8 posterior patients) or tumour (n = 36; 25 frontal, 11 posterior patients). Importantly, we have previously shown that the grouping together of patients with different aetiologies for the purposes of examining cognitive variables is methodological justifiable (Cipolotti et al., 2015).

In addition, 39 HCs with no history of neurological or psychiatric disorders were included for comparison. The study was approved by the National Hospital for Neurology and Neurosurgery & Institute of Neurology Joint Research Ethics Committee and written informed consent was obtained from all participants.

### 2.2 Procedure

All patients and HCs were assessed on a battery of baseline neuropsychological tests, a fluid intelligence test and the CET-A (n = 22)). All tumour patients, with the exception of 4 patients, were assessed after tumour resection (mean = 54.90 days, SD = 131.53). Stroke patients were assessed on average after 284.25 days (SD = 588.43) from their acute neurological event.

### 2.3 Neuroimaging investigation

Of the 34 frontal patients, 22 patients had available MRI scans that were available for lesion analyses. All but for two FLAIRs were T2-weighted sequences with a typical pixel resolution of .47 by .47 by 6.5, acquired for clinical purposes on a variety of scanners. The four patients who had not undergone tumour resection were excluded from analyses due to the poor definition of the lesion boundary. All scans were reviewed by an independent neurologist (PN) who was blind to the medical history and neuropsychological performance of each patient. The abnormal areas of each brain image were segmented and transformed into Montreal Neurological Institute (MNI) stereotactic space with the aid of the following semi-automated procedure, which is related to a well-established approach for delineating brain lesions (Seghier et al., 2008) based on SPM12 (http://www.fil.ion.ucl.ac.uk/spm/software/spm12/). First, MIPAV's livewire tool (https://mipav.cit.nih.gov/) was used manually to create a rough binary lesion mask. The MR volume was clamped within the interval of .01–.99 of the cumulative signal distribution, estimated with kernel density estimation (Botev et al., 2010). Both the MR volume and the lesion mask were then resampled to 1 mm^3^ isotropic with 4th degree spline interpolation. The MR volume was then processed through SPM12's unified segmentation/normalization procedure with all parameters set at default except for the use of a non-parametric mixture model. The estimated deformation field was used to transform the rough lesion mask into MNI space. The inverse deformation field was used to transform SPM12's standard brain mask image into the MR volume's native space. After smoothing with a Gaussian kernel of 3 mm FWHM, this brain mask was applied to the original, native-space MR volume to remove all tissue outside the brain. This skull-stripped version of this volume was then passed through SPM12's unified segmentation/normalization procedure, this time using a different set of probability distributions for each tissue type. The first three - for grey matter, white matter, and cerebrospinal fluid - were as standard. Since the image is skull-stripped, the fourth distribution was supplied as the sum of the non-brain tissue compartments in SPM12's tissue probability 4D volume (i.e., volumes 4–6). The fifth probability distribution was the normalized version of the rough lesion mask, smoothed with a Gaussian kernel of 8 mm^3^ isotropic. The deformation field derived from this procedure was used to transform the thereby estimated fifth (i.e., lesion) compartment into MNI space. This was smoothed with a Gaussian kernel of 6 mm^3^ isotropic and the segmentation/normalization procedure was run a third time, now using this updated lesion distribution. Note that since smoothing was applied only to empirical lesion distributions at the start of the procedure and during intermediate stages of the algorithm, not to the source images themselves, the final lesion segmentation will be of comparable smoothness to standard tissue segmentations for MR images of this quality. At the conclusion of this procedure, the final deformation field was used to transform both the original image and the final estimate of the lesion compartment, thresholded at a probability of .9, into standard MNI space. These compartments were used to quantify lesion volume. The resulting segmented lesion was checked slice-by-slice by a neurologist with extensive expertise in lesion mapping for accuracy (PN). A mean image of all normalized volumes was created for visualization purposes, together with a sum of the estimated lesion distributions, all resliced to 1.5 mm^3^ isotropic resolution.

### 2.4 Baseline neuropsychological assessment

All patients and HCs had a single neuropsychological assessment comprising well-known tests with published standardised normative data. All tests were administered and scored according to the published manual. Premorbid level of optimal functioning was estimated using the National Adult Reading Test (NART; Nelson and Willison, 1991). Arithmetical skills were assessed using the Graded Difficulty Arithmetic Test (GDAT; Jackson and Warrington, 1986). Naming ability was assessed using the Graded Naming Test (GNT; McKenna and Warrington, 1980). General knowledge was assessed using the Information subtest from the Wechsler Adult Intelligence Scale-III (WAIS-III; Wechsler, 1997).

### 2.5 Fluid intelligence – Raven test

Fluid intelligence was assessed using Raven's Advanced Progressive matrices (RAPM). This is an untimed, relatively culture free, non-verbal test of abstract reasoning (Arthur and Day, 1994). Raw scores were converted to scaled scores using available standardized norms.

### 2.6 Cognitive estimation task (CET)

The 9-items of the CET-A were administered to all HCs. The estimation questions included questions such as, “*What is the length of the average new born baby?”* or “*What is the maximum speed of a cheetah*?”. All questions required numerical responses. As per standardized procedures, participants were told that for most questions there was no exact answer or it was unlikely they would know the answer. Participants were requested to provide a reasonable guess or estimate of what the answer would be. The estimation questions were asked out loud by the experimenter and participants gave their answers orally. Participants could answer the items using their preferred unit of measurement but, when scoring the items, the responses were converted to the same unit of measurement. This was to ensure that participants did not fail to provide an appropriate estimate due to unfamiliarity with the unit of measurement rather than poor estimation abilities. Participants were given as much time as necessary to produce estimations. For each item, participants were asked if they were sure that the response they had provided was a reasonable estimate and, if not, they were able to change their response.

Following the scoring procedures described by MacPherson et al. (2014), each response was awarded a score of 0, 1, 2 or 3, resulting in possible scores ranged from 0 (best performance) to 27 (worst performance). A score of zero was attributed to answers considered *normal*. These responses fell between the 20th and above the 80th percentile of the published normative data. A score of 1 was attributed to answers considered *quite extreme*. These responses were between the 10th and the 20th percentiles or between the 80th and 90th percentiles. A score of 2 was attributed to answers considered *extreme*, which were between the 5th and the 10th percentiles or between the 90th and 95th percentiles. Lastly, a score of 3 was attributed to answers considered *very extreme*. These responses were below the 5th percentile or above the 95th percentile. The overall raw scores were then adjusted for age, gender and education according to the correction grids in MacPherson et al. (2014).

In our subsequent analyses, we considered the adjusted overall scores and three error measures following the procedure devised by Shallice and Evans (1978). Measure I comprised responses rated as *quite extreme* (i.e., the percentage of items scored 1), measure II comprised *extreme and very extreme* responses (i.e., the percentage of items scored 2 or 3) and measure III comprised *very extreme* responses only (i.e., the percentage of items scored as 3).

#### 2.6.1 Statistical analysis

The statistical analyses were carried out using IBM SPSS Statistics 22 (http://www01.ibm.com/software/analytics/spss/). One-way analysis of variance (ANOVA) was used to investigate whether the frontal, posterior patients and HCs significantly differed in terms of age, years of education, NART IQ, and performance on the baseline neuropsychological tests and fluid intelligence measures. To adjust for multiple comparisons, the p-values were Bonferroni corrected. A chi-square test was used to investigate whether there was a significant difference in terms of the gender ratio. Independent samples *t*-tests were used to investigate whether there were differences in demographic variables and cognitive performance between left and right frontal patients and between left and right posterior patients. Levene's test was used to assure equality of variances.

One-way ANOVAs were conducted to investigate whether performance on the CET A overall adjusted scores significantly differed between the frontal, posterior patients and HCs. To adjust for multiple comparisons, results were Bonferroni corrected (.05/2 = .025). When significant results were found, analyses of covariance (ANCOVA) were conducted, entering GDAT and RAPM as covariates.

To investigate whether there were significant differences between patients and HCs on the three error measures of the CET-A, we ran ANCOVAs with age and education as covariates. When the results were significant, we conducted additional ANCOVAs with age, education, GDAT and RAPM as covariates.

Pearson's product moment correlational analyses were also conducted to examine whether there was a significant relationship between CET-A performance and fluid intelligence in our frontal patients.

For the imaging analysis, the lesion volume of the left frontal (n = 8) and right frontal (n = 10) group was compared using one-way ANOVA. Pearson's product moment correlational analysis was conducted to examine whether there was a significant relationship between lesion volume and performance on CET-A.

## 3 Results

### 3.1 Demographic and baseline neuropsychological assessment

Table 1 show the means and standard deviations for patients and HCs for the demographic and neuropsychological measures. The frontal and posterior patients and HCs did not significantly differ in terms of their age (p > .1), gender (p > .1), years of full-time education (p=.076) or NART IQ (p > .1; see Table 1). The left and right frontal patients did not significantly differ in terms of age (p = .385), gender (*p* = .424), years of education (p = .435) or NART IQ (p = .457). Similarly, the left and right posterior patients did not significantly differ in terms of age (*p* = .287), gender (*p* = .746), years of education (*p* = .965) or NART IQ (*p* = .663).

**Table 1:** Means and standard deviations for the demographic and baseline neuropsychological data.

	**Frontal Patients (N = 34)**	**Posterior Patients (N = 19)**	**Healthy Controls (N = 39)**
Time between damage and assessment (days)	246.53(614.88)	152.86(197.25)	N/A
Age (years)	49.85	51.58	54.59
	(15.70)	(15.46)	(4.70)
Gender (Male/Female)	20/14	11/8	16/23
Education (years)	14.79	12.79	13.85
	(3.00)	(3.24)	(3.05)
NART IQ	111.84	108.21	112.79
	(9.03)	(9.55)	(8.05)
GDAT (max = 24)	13.95(5.22)	9.33**(5.50)	16.00(5.32)
GNT (max = 30)	21.39**	20.78**	24.36
	(4.33)	(3.98)	(3.16)
Information subtest (max = 28)	21.50	19.20	22.79
	(6.38)	(5.18)	(3.73)
RAPM (max = 12)	9.05(2.66)	7.22(2.22)	8.56(1.98)

* = p < .05, ** = p < .01, *** = p < .001 compared to healthy controls.

NART = National Adult Reading Test; GDAT = Graded Difficulty Arithmetic Test; GNT = Graded Naming Test; RAPM = Ravens Advanced Progressive Matrices.

There was a significant difference between frontal and posterior patients and HCs in performance on the GDAT (F(1, 66) = 5.92, p = .004). Bonferroni post-hoc analyses revealed a significant difference between posterior patients and HCs (p = .004). In contrast, there were no significant differences between frontal and HCs (p = .478) or frontal and posterior patients (p = .098). There were also no significant differences between vascular and tumour patients performing the NART (p = .483), GDAT (p = .658) or GNT (p = .779).

There was a significant difference between frontal and posterior patients and HCs in performance on the GNT (F(1, 88) = 7.93, p = .001). Bonferroni post-hoc analyses revealed a significant difference between frontal patients and HCs (p = .005) and posterior patients and HCs (p = .004). There was no significant difference between frontal and posterior patients (p = .999).

There were no significant differences between frontal and posterior patients and HCs on the Information subtest from the WAIS-III (*p* = .113). There were also no significant differences between vascular and tumour patients on the Information subtest from the WAIS-III (p = .468).

### 3.2 Fluid intelligence

A one-way ANOVA revealed no significant differences in performance between frontal and posterior patients and HCs on the RAPM, a test of fluid intelligence (F(1, 66) = 2.11, p = .130).

There were no significant differences between the left and right frontal patients or the left and right posterior patients on the RAPM (*p* = .475 and *p* = .238, respectively).

### 3.3 Cognitive estimation task (CET)- overall adjusted score

**Fig. 1:**
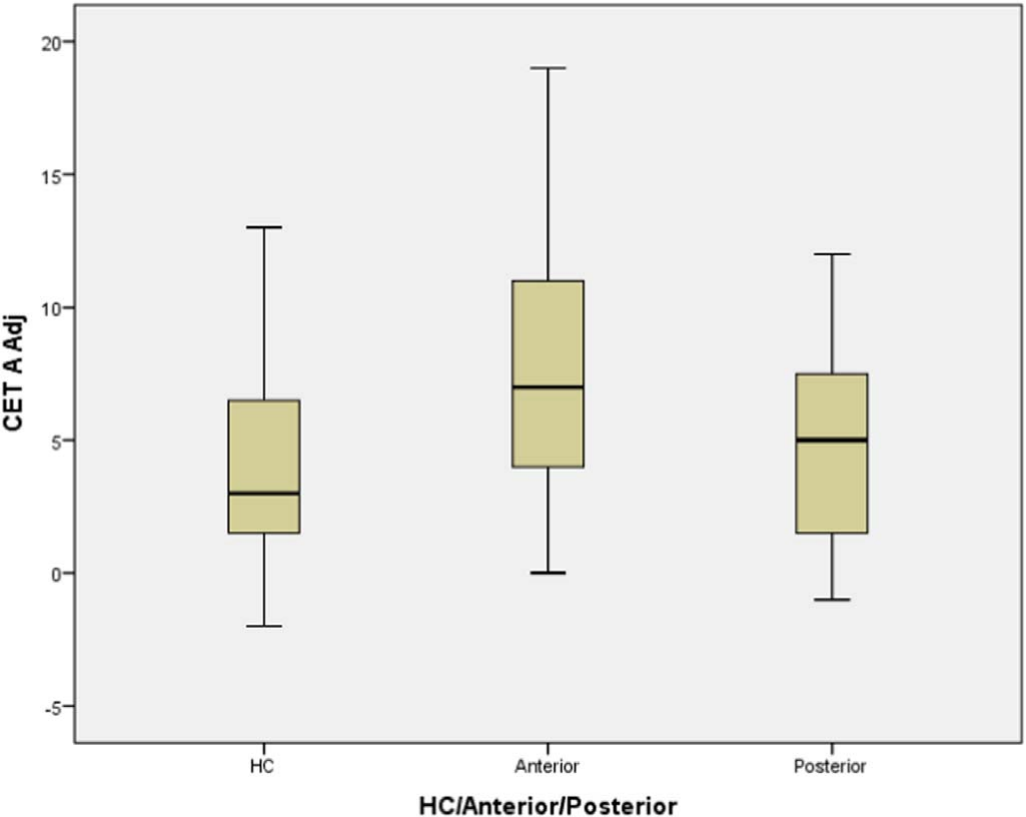
Boxplot displaying performance on CET-A Adjusted scores of Frontal, Posterior and HC.

Table 2 show the means and standard deviations for patients and HCs performing the CET-A. Fig. 1 show the group data in box plots. A one-way ANOVA showed a significant difference between the three groups (F(1, 89) = 10.380, p < .001). Post-hoc analysis revealed that the frontal patients had significantly higher overall error scores than HCs (p < .001) and posterior patients (p = .024). There was no significant difference between the posterior patients and HCs (p = .999).

**Table 2:** Mean and standard deviations for the CET-A overall adjusted error scores and the percentage of quite, extreme and very extreme error scores.

	Frontal Patients	Posterior Patients	Healthy Controls
*Overall adjusted score*	7.94***(4.76)	4.74^a^^,^*(3.97)	3.62(3.54)
*Error Measures*			
Measure I - Quite extreme only	14.24(9.87)	21.57(14.94)	14.81(11.77)
Measure II - Extreme and very extreme	30.82***(19.82)	18.30(14.67)	15.95(13.68)
Measure III - Very extreme only	20.49***(17.42)	8.50^a^^,^**(10.78)	6.84(10.07)

**Bold** indicates significant difference from healthy controls.

* = p < .05.

** = p < .01.

*** = p < .001.

a = Indicates significant difference of frontal versus posterior patients.

We also investigated whether the difference between the frontal patients and HCs remained once performance on arithmetic and fluid intelligence tasks were controlled for. We ran an ANCOVA with GDAT and RAPM as covariates. While GDAT was found to be a significant covariate (p = .037), RAPM was not (p = .410). The ANCOVA result indicated that a significant difference remained between the frontal patients and HCs (F(1, 55) = 8.70, *p* = .005). We found no significant difference between left and right frontal patients on overall CET-A scores (*p* = .997). Additionally, we found no significant differences between vascular and tumour patients in their overall CET-A scores (*p =* .928). 12 out of the 34 frontal patients were impaired on the CET A, achieving an adjusted error score of 10 or more. 3 out of the 19 posterior patients were impaired on the CET A and none of the HC.

### 3.4 CET- A – three error measures

Table 2 shows the mean percentage and standard deviations for the responses scored as *quite extreme*- Measure I-, the responses scored as *extreme and very extreme*- Measure II-, and the responses scored as *very extreme*- Measure III-, in patients and HCs.

ANCOVAs with age and education as covariates revealed no significant differences (F(2, 83) = 2.30*, p* = .107) between patients and HCs in the percentage of *quite extreme* responses. In contrast, we found significant differences for the percentage of *extreme and very extreme* responses (F(2, 82) = 7.51*, p* = .001) and the percentage of *very extreme* responses (F(2, 83) = 10.73*, p* < .001) between the patient groups and HCs. For the percentage of *extreme and very extreme* responses, Bonferroni post-hocs revealed a significant difference between frontal patients and HCs (*p* = .001). In contrast, there was no significant difference between frontal and posterior patients (p = .057) and posterior patients and HCs (p = .99). For the percentage of *very extreme* responses only, Bonferroni post-hoc *t*-tests revealed a significant difference between frontal patients and HCs (*p* < .001) and frontal and posterior patients (p = .008). In contrast, there was no significant difference between posterior patients and HCs (p = .99).

An ANCOVA with age, education, GDAT and RAPM entered as covariates revealed no significant difference for the percentage of *quite extreme* responses in frontal patients and HCs (F(1, 53) = .069*, p* = .794). However, significant differences were found for *extreme and very extreme* responses and *extreme responses* only in frontal patients and HCs (F(1, 53) = 5.99*, p* = .018 and F(1, 53) = 12.18*, p* = .001 respectively).

### 3.5 CET-A – correlational analysis

First, two-tailed Pearson's correlational analyses were conducted to investigate whether there was a relationship between performance on CET-A and fluid intelligence in frontal patients. No significant correlation was found (r = −.125, *p* = .589). Secondly, we investigated whether there were relationships between the three CET-A error measures and fluid intelligence. Again, we found no significant correlations: *quite extreme* (r = −.131, *p* = .571); *extreme and very extreme* (r = −.172, *p* = .456), and *very extreme* only (r = −.154, *p* = .505).

### 3.6 Neuroimaging results

**Fig. 2:**
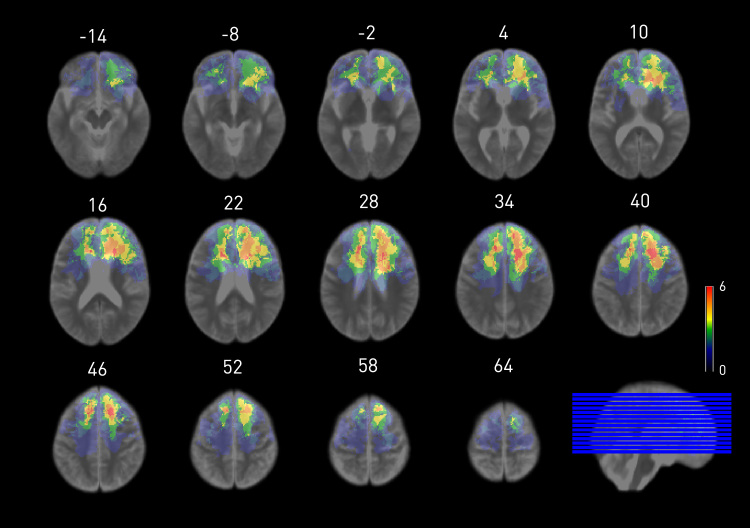
**Axial slice renders of the lesion distributions.** The underlay is the mean of all MR volumes and the overlay is the sum of the thresholded lesion masks, all transformed into MNI space, resliced to 1.5 mm^3^ isotropic resolution, and displayed in neurological convention. The vertical dimension is given above each slice. Visualized with Micron (http://people.cas.sc.edu/rorden/mricron/index.html).

**Fig. 3:**
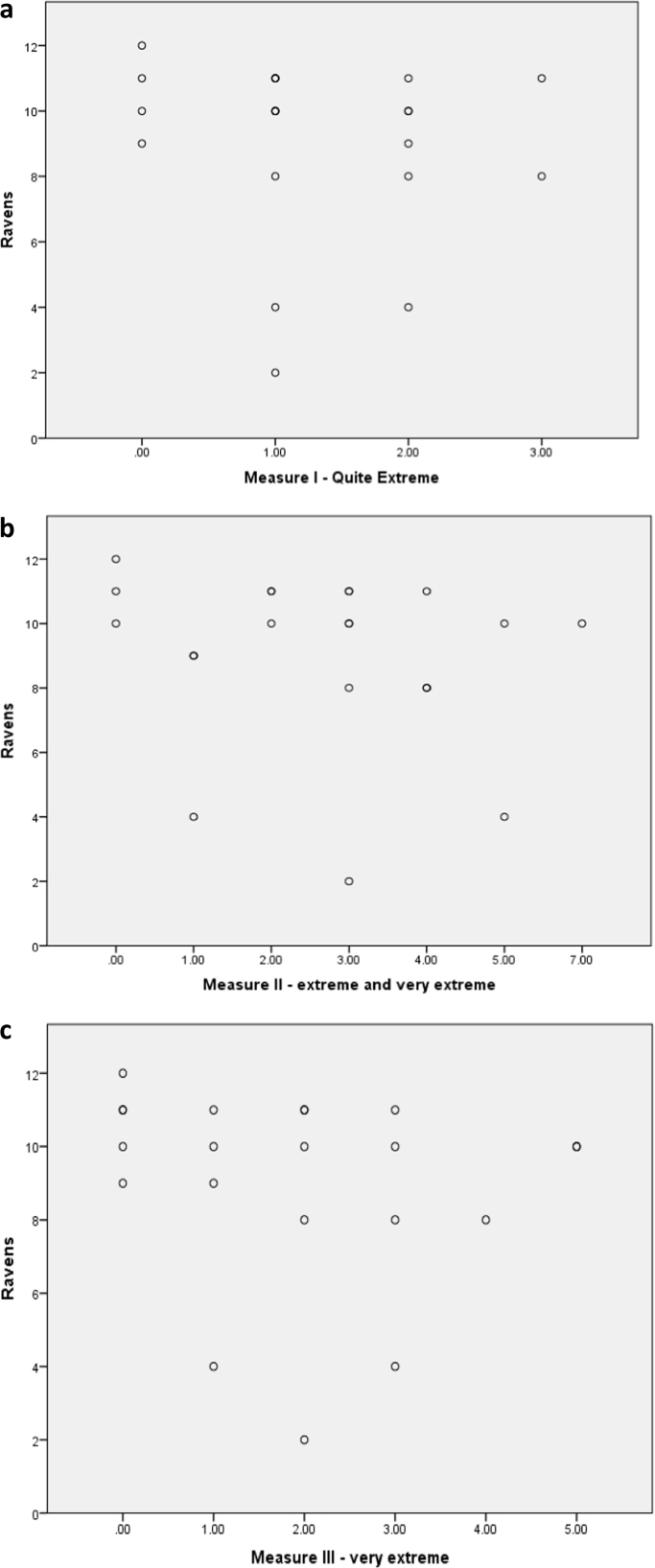
a. Scatter Plot displaying correlation between Measure I –Quite Extreme responses and fluid intelligence (Ravens) in Frontal patients. b. Scatter Plot displaying correlation between Measure II –extreme and very extreme responses and fluid intelligence (Ravens) in Frontal patients. c. Scatter Plot displaying correlation between Measure III –very extreme responses and fluid intelligence (Ravens) in Frontal patients.

Given that we found CET-A was sensitive to detecting impairment in frontal patients compared with both HCs and posterior patients, we further investigated whether performance was related to frontal lesion volume or location in a subset of 18 patients with available MRI scans. We found no significant differences between this subset of patients and our larger frontal sample on age, education, gender or performance on the CET-A (p > .05). We found no significant relationship between lesion size and performance on the CET-A overall adjusted score (*p* = .980) or in Error Measure III, extreme and very extreme errors (*p* = .718). There was no significant difference in average lesion size between the left (n = 8) and right (n=10) frontal groups (Left *M* = 43.39 mm^3^, SD = 34.10; Right *M* = 63.43 mm^3^, SD = 51.89; *p* = .362). We noted that the three patients who made more than 50% Measure III errors in their responses on CET-A all had right-sided lesion. The degree of overlap between lesions together with the relatively small sample precludes precise anatomical inferences from the imaging data. We therefore simply present the lesion distributions in Fig. 2, overlaid on the mean group image (Fig. 3).

## 4 Discussion

To our knowledge, our study is one of the few investigations on the performance of patients with focal frontal or posterior lesions and HCs on the CET. Our analyses allowed us to address the effect of frontal lobes lesions in our newly developed CET-A. The results revealed that frontal patients provided significantly higher overall adjusted scores than posterior patients and HCs for CET-A. Importantly, our frontal patients’ impairment cannot be explained by dysphasia, as our frontal patients did not present with significant nominal impairments when compared to posterior patients. Moreover, our frontal patients did not significantly differ from HCs in terms of their years of education, NART IQ, general knowledge or calculation performance and yet they produced significantly higher CET error scores. Interestingly, the posterior patients were more impaired than the frontal patients on the GDA, yet they produced fewer errors than the frontal patients on the CET A. It is also important to note that, for the CET-A, the significant difference in performance compared to HCs remained significant when performance on arithmetic and fluid intelligence measures were taken into account. This suggests that CET-A impairment in frontal patients cannot be explained by calculation or fluid intelligence deficits. Good language, general knowledge, calculation skills and fluid intelligence may be necessary but not sufficient for normal CET performance. Our findings suggest that frontal-related executive strategic processes are needed in order to obtain normal scores on the CET.

The impaired CET A performance of our frontal patients when compared to HCs demonstrates that our CETA is affected by frontal lobe damage. This supports our previous findings in MacPherson et al. (2014). One limitation of our previous study was the lack of the inclusion of a posterior control group. In order to provide evidence for frontal lobe localisation of CET processes, we argued for the need to investigate whether patients with non-frontal lesions are able to produce appropriate cognitive estimates on our new CET compared to HCs.

The significant difference in the performance of frontal and posterior patients in CET-A and the lack of difference between the posterior patients and HC indicates that impairments on this test are restricted to frontal lesions. Thus, our results suggest that CET A is suitable for assessing frontal lobe dysfunction in clinical practice and research. Moreover, as reported above, CET-A impairments in our frontal patients cannot be entirely explained by impairments in fluid intelligence, as controlling for fluid intelligence using the RAPM did not remove the significant differences. Investigation of the relationship between performance on a fluid intelligence test (RAPM) and the CET-A revealed that there were no significant correlations between performance on the RAPM and CET-A. Roca et al. (2010) have previously reported that fluid intelligence is a substantial contributor to frontal patients’ executive impairments. In contrast, Cipolotti et al. (2016) have recently demonstrated that frontal patients’ impairment on the Stroop test and the suppression part of the Hayling Sentence Completion test cannot be fully explained by fluid intelligence. Our current findings demonstrate that the CET-A is another ‘executive’ task for which impairment cannot be accounted for entirely by fluid intelligence abilities.

D’Aniello et al. (2015) have proposed that the CET is a measure of crystallized abilities rather than fluid abilities. Indeed, previous research has reported that performance on tests of crystallized intelligence, such as vocabulary, reading ability and general knowledge, are related to CET performance (Della Sala et al., 2003, MacPherson et al., 2014, O’Carroll et al., 1994). However, both our previous work (MacPherson et al., 2014) and the current study demonstrate that frontal patients’ CETA impairments exist even when they are matched with HCs in terms of crystallized abilities. Therefore, while fluid and crystallized abilities do contribute to CETA performance, it is unlikely these processes can entirely account for CETA performance.

We analysed the patients’ and HCs’ responses in terms of the percentage of their errors that were responses rated as *quite extreme, extreme and very extreme* and *very extreme* only. For CET-A, frontal patients had a significantly higher percentage of *extreme and very extreme* and *very extreme only* responses when compared to HCs but not when compared to posterior patients. However, for the *very extreme* only responses, frontal patients were significantly impaired when compared to posterior patients and HCs. These differences remained significant when age, education, GDAT and RAPM were accounted for. Moreover, fluid intelligence did not correlate with the percentage of *extreme* and *very extreme* responses provided by our frontal patients on CET-A.

Overall, the error analyses results suggest that *extreme* and *very extreme* responses are affected by frontal lobe damage. However, only for CET-A *very extreme* responses do patients with frontal lobe damage perform worse than posterior patients. In addition, for the *very extreme responses* posterior patients do not differ significantly from HC. Thus, for CET-A, the *very extreme* responses only represent a selective measure of frontal “executive” impairment, in addition to overall performance. Notably, our error analyses provide a source of behavioral evidence, not available from neuroimaging methodologies involving healthy participants that allow us to speculate about the type of function damaged. *Very extreme* responses are likely to arise from impairments in generating the appropriate strategy for answering the question or in error monitoring. Future research is needed to explore further the frontal executive function that is impaired in patients with cognitive estimation deficits.

We also found that the CET-A overall adjusted score or Error Measure III was not related to lesion size. Statistically we found no difference in the performance on CET A between right and left frontal patients. Of course, it remains possible that the lack of a lateralization effect may be due to the relatively small size of our frontal patient sample. In this context we noted that Measure III errors were more prominent in right rather than left frontal patients. Future studies with larger sample of frontal patients are needed to address the question as to whether specific prefrontal areas are critically involved in cognitive estimation.

To the best of our knowledge, no neuroimaging studies have examined the neural correlates of CET performance and/or estimation abilities involving quantity, length, speed etc. Instead, there is a large neuroimaging literature that has focused on time estimation (for a review see Coull et al., 2011). Time estimation abilities have been associated with activation of the inferior frontal gyrus, as well as other brain regions. However, the processes involved in time estimation are clearly different to those involved in the CET, so it remains unknown whether this frontal activation would also be reported during the CET. For now, only lesion studies involving patients have provided information about the functional architecture of the CET.

In conclusion, our findings suggest the CET-A is a useful test to assess executive functions. Both the overall performance and the *very extreme responses* on this test have been shown to be affected by and restricted to frontal lobe damage. Fluid intelligence is not a substantial contributor to cognitive estimation impairment. Hence, our frontal patients’ cognitive estimate impairment cannot easily be accounted for by a general, common frontal process such as fluid intelligence. Our investigation provided further support for the utility of neuropsychological studies for examining executive functions by indicating that the frontal lobes are critical for the generation of cognitive estimates. It also illustrate that neuropsychological approach provide additional, meaningful, behavioral evidence, namely the type of errors made, not usually available from other cognitive neuroscience methods, (see Shallice and Cipolotti, 2017, for further discussion).
