# Supplementary material for: Cognitive estimation: Performance of patients with focal frontal and posterior lesions
Source: Neuropsychologia. 2018 Jul 1;115:70–7. doi: 10.1016/j.neuropsychologia.2017.08.017 (PMC6018564; doi:10.1016/j.neuropsychologia.2017.08.017)
Supplement: Supplementary file 1 — Supplementary material [file mmc1.docx]

**Cognitive Estimates Test -Version A**

1. What is the maximum speed of a Harley-Davidson motorbike (km/h)?
2. What is the length of the average newborn baby (cm)?
3. How fast do race horses run (km/h)?
4. What is the average jogging speed (km/h)?
5. How many segments are there in an orange?
6. What is the length of a new pencil (cm)?
7. What is the maximum speed of a cheetah (km/h)?
8. What is the length of an average men’s mountain bike (m)?
9. How many keys are there on a standard computer keyboard?
